# Clinic-based diabetes screening at the time of HIV testing and associations with poor clinical outcomes in South Africa: a cohort study

**DOI:** 10.1186/s12879-021-06473-1

**Published:** 2021-08-10

**Authors:** Rachel W. Kubiak, Mario Kratz, Ayesha A. Motala, Sean Galagan, Sabina Govere, Elisabeth R. Brown, Mahomed-Yunus S. Moosa, Paul K. Drain

**Affiliations:** 1 Department of Epidemiology, Health Sciences Building, University of Washington, Seattle, WA USA; 2 Cancer Prevention Program, Division of Public Health Sciences, Fred Hutchinson Cancer Research Center, Seattle, WA USA; 3 Department of Medicine, University of Washington, Seattle, WA USA; 4 Department of Diabetes and Endocrinology, University of KwaZulu-Natal, Durban, South Africa; 5 Department of Global Health, University of Washington, Seattle, WA USA; 6 AIDS Healthcare Foundation, Durban, South Africa; 7 Statistical Center for HIV/AIDS Research and Prevention, Fred Hutchinson Cancer Research Center, Seattle, WA USA; 8 Department of Biostatistics, University of Washington, Seattle, WA USA; 9 Department of Infectious Diseases, University of KwaZulu-Natal, Durban, South Africa

**Keywords:** Diabetes mellitus, HIV, Hyperglycemia, Pre-diabetes, Mortality, Tuberculosis

## Abstract

**Background:**

HIV clinical care programs in high burden settings are uniquely positioned to facilitate diabetes diagnosis, which is a major challenge. However, in sub-Saharan Africa, data on the burden of diabetes among people living with HIV (PLHIV) and its impact on HIV outcomes is sparse.

**Methods:**

We enrolled adults presenting for HIV testing at an outpatient clinic in Durban. Those who tested positive for HIV-infection were screened for diabetes using a point-of-care hemoglobin A_1c_ (HbA_1c_) test. We used log-binomial, Poisson, and Cox proportional hazard models adjusting for confounders to estimate the relationship of diabetes (HbA_1c_ ≥ 6.5%) with the outcomes of HIV viral suppression (< 50 copies/mL) 4–8 months after antiretroviral therapy initiation, retention in care, hospitalization, tuberculosis, and death over 12 months.

**Results:**

Among 1369 PLHIV, 0.5% (n = 7) reported a prior diabetes diagnosis, 20.6% (95% CI 18.5–22.8%, n = 282) screened positive for pre-diabetes (HbA_1c_ 5.7–6.4%) and 3.5% (95% CI 2.7–4.6%, n = 48) for diabetes. The number needed to screen to identify one new PLHIV with diabetes was 46.5 persons overall and 36.5 restricting to those with BMI ≥ 25 kg/m^2^. Compared to PLHIV without diabetes, the risk of study outcomes among those with diabetes was not statistically significant, although the adjusted hazard of death was 1.79 (95% CI 0.41–7.87).

**Conclusions:**

Diabetes and pre-diabetes were common among adults testing positive for HIV and associated with death. Clinic-based diabetes screening could be targeted to higher risk groups and may improve HIV treatment outcomes.

## Introduction

Diabetes is a chronic non-communicable disease that increases the risks of infection, hospitalization, and death and remains a major cause of global morbidity and mortality [1, 2]. In sub-Saharan Africa, the age-adjusted burden of diabetes is estimated to be 15.5 million people (4.4%) and is projected to reach 40.7 million people by the year 2045 [2]. As antiretroviral therapies (ART) have increased life expectancy for people living with HIV (PLHIV), diabetes may play a larger role in chronic care and management. Among PLHIV, diabetes is common and associated with age and long-term HIV or ART exposure [3, 4].

In sub-Saharan Africa, where the burden of diabetes and HIV are high, there is limited data on the prevalence of diabetes among PLHIV and the impact of diabetes on clinical outcomes. Available data suggest PLHIV with diabetes may be at increased risk of active tuberculosis (TB) and death compared to PLHIV without diabetes [5–7].

A major challenge for diabetes care is early diagnosis [2, 8, 9]. In South Africa, the country-wide diabetes prevalence is 5.4% and more than twice that in urban settings [10, 11]. However, prevalence surveys show 31–85% of people with diabetes are unaware of their disease status [9, 11, 12]. Clinics have established infrastructure for HIV screening and are uniquely positioned to facilitate diabetes diagnosis and treatment, but diabetes screening rates are thought to be low [13, 14].

We examined the burden of diabetes, pre-diabetes, and associated risk factors among adults testing positive for HIV in South Africa. We estimated the number needed to screen (NNS) overall and using age and/or body mass index (BMI) to guide testing of patients at high risk for diabetes, and assessed relationships between diabetes and key clinical outcomes.

## Methods

### Study population

Beginning in September 2013, we enrolled adults presenting for outpatient HIV screening at the iThembalabantu People’s Hope Clinic, a large public clinic in the Umlazi township of Durban, South Africa. Eligible participants were ≥ 18 years, ART-naïve, English or Zulu speaking, had not received anti-fungal therapy within three months, and were not pregnant. For these analyses, we included only HIV-positive participants from January 2017 to February 2019 when point-of-care hemoglobin A_1c_ (HbA_1c_) testing for diabetes was performed routinely.

### Ethics approval and consent to participate

All study participants provided written informed consent in English or Zulu. The study was approved by the University of KwaZulu-Natal Biomedical Research Ethics Committee (BF052/13) and the University of Washington Institutional Review Board (49563).

### Study procedures

Prior to HIV testing, a research assistant collected demographic (age, sex, ethnicity, education, measures of socioeconomic status) and health information (cigarette and alcohol use, history of diabetes, kidney disease, liver disease) using a standardized in-person questionnaire. Research assistants also measured the participant’s blood pressure, height, and weight. Serial HIV rapid testing and referral for ART was performed at the clinic by routine clinic staff as per local standard of care [15].

Among PLHIV, a research nurse obtained blood samples, screened participants for TB, and performed a fingerpick HbA_1c_ test (A1cNow®^+^, PTS Diagnostics, Indianapolis, IN). A1cNow® + is an immunoassay that has been certified by the National Glycohemoglobin Standardization Program (NGSP), which works to harmonize HbA_1c_ tests [16]. Participants were referred to a clinician for additional testing and care according to national guidelines [17].

At three, six, and 12 months after enrollment, a research assistant reviewed pharmacy and medical records and attempted to telephone the participant at least three times. At each time point they documented available HIV outcomes including HIV viral load, hospitalizations, and death. Hospitalized participants had additional chart review to determine the cause. Opportunistic infections were documented including herpes simplex lesions, oral/esophageal candida, pneumonias, cytomegalovirus, cryptosporidiosis, bacterial meningitis, toxoplasmosis, and herpes zoster. Tuberculosis diagnoses were confirmed in the South African TB Registry. Death was determined through clinical record review, call to a named contact of the participant if they could not be reach directly, and review of the South African National Death Registry for participants who could not be contacted, had no recent medical records, and were not confirmed to be attending another clinic. Participants were considered retained in care if they had a 12 month study visit or were known to have transferred to another study clinic. They were considered lost to follow-up if they did not have a 12 month clinic or study visit and were not known to have transferred to another clinic or died.

### Study definitions

We defined diabetes as HbA_1c_ ≥ 6.5%, which is consistent with World Health Organization (WHO) and American Diabetes Association (ADA) guidelines for lab-based high-performance liquid chromatographic HbA_1c_ testing [18, 19]. We considered pre-diabetes to be HbA_1c_ of 5.7–6.4% and hyperglycemia to be inclusive of both diabetes and pre-diabetes using HbA_1c_ ≥ 5.7% [19]. Measured cofactors for diabetes included BMI (overweight 25–29.9 kg/m^2^; obese ≥ 30 kg/m^2^), mean arterial pressure (MAP, [(2*diastolic blood pressure) + systolic blood pressure]/3), and anemia (hemoglobin < 12 g/dL in women or < 13 g/dL in men) [20]. We used the nine-question validated Household Food Insecurity Access Scale (HFIAS) to ascertain food insecurity and defined it as the presence or absence of any food insecurity [21]. HIV viral load suppression was defined as ≤ 50 copies/mL 4–8 months after ART initiation. Tuberculosis was defined as acid-fast bacilli smear positive, GeneXpert positive, culture positive, or empiric treatment initiation.

### Statistical analysis

We performed statistical analyses using SAS version 9.4 (Cary, NC). We used the Agresti-Coull method to calculate 95% confidence intervals (CI) for the prevalence of diabetes and pre-diabetes [22]. We tested for correlates of diabetes at baseline and the intermediate study outcomes of ART initiation, missing ≥ 1 clinic visit, and missing ≥ 1 ART pharmacy refill using the χ-square or Fisher’s exact test for categorical variables and ANOVA for continuous variables.

We estimated the NNS to yield one case of diabetes or hyperglycemia, the proportion identified, and proportion missed overall and for a range of age and BMI cut points. We generated receiver operating characteristic (ROC) curves using logistic regression to estimate the area under the curve (AUC) and identified the value at which sensitivity and specificity were maximized using Youden index [23].

We estimated the risk of key HIV clinical outcomes by diabetes status in univariate analysis and after adjusting for age, sex, BMI, ART initiation, opportunistic infections, and anemia. We used log-binomial regression or Poisson regression with robust standard errors to estimate the relative risk (RR) of (1) not achieving viral suppression, (2) retention in care, and (3) ART default or lost to follow-up. We used Cox proportional hazard models to estimate the relative difference in time to (1) first hospitalization, (2) tuberculosis diagnosis, or (3) death.

## Results

### Description of study population

Of the 7,877 adults who presented to iThembalabantu Clinic for HIV screening and were provisionally enrolled into the study, 3,104 tested positive for HIV. Hemoglobin A_1c_ testing following a positive HIV test was performed as part of routine study procedures for 1,207 participants. The majority (52%) of participants were diagnosed with HIV at the time of enrollment. Those with a diagnosis prior to study enrollment reported their diagnosis occurred a median of nine months prior (IQR 0–43.5 months) and 12% reported previously receiving ART. Overall, 44% of participants were men, mean age was 31.3 ± 9.5 years, and median CD4 count was 353 cells/mm^3^ (IQR 209–545 cells/mm^3^). Anemia was common among both men (31%) and women (54%). Only 1.3% (n = 7) reported a prior diagnosis of diabetes. A viral load measurement was available for 806 participants collected 4–8 months after ART initiation (mean 6 months, standard deviation 0.9) at which point 83% (n = 668) were virally suppressed. At 12-months, 51% of participants were reached by phone call.

### Correlates of diabetes and pre-diabetes at time of HIV testing

Overall, 2.2% (95% CI 1.4–3.2%, n = 26) of participants screened positive for diabetes and 17.7% (95% CI 15.6–19.9%, n = 213) for pre-diabetes (Table 1). The mean age of participants who screened positive for diabetes was higher than those with pre-diabetes or normal HbA_1c_ (p < 0.001).

**Table 1:** Baseline characteristics of people living with HIV screened for diabetes in South Africa

	Overall	HbA_1c_ ≤ 5.6%	HbA_1c_ 5.7–6.4%	HbA_1c_ ≥ 6.5%	p-value
	(n = 1207) N (%) or mean ± SD	(n = 968) N (%) or mean ± SD	(n = 213) N (%) or mean ± SD	(n = 26) N (%) or mean ± SD	
Sociodemographics					
Male	536 (44.4)	430 (44.4)	94 (44.1)	12 (46.2)	0.981
Age (years)	31.3 ± 9.5	32.5 ± 9.0	35.1 ± 10.5	43.0 + 11.4	** < 0.001**
Zulu or Xhosa (n = 1205)	1187 (98.5)	954 (98.8)	208 (97.7)	24 (96.2)	0.151
Married	63 (5.2)	44 (4.6)	17 (8.0)	2 (7.7)	0.106
Completed high school or higher degree	733 (60.7)	588 (60.7)	133 (62.4)	12 (46.2)	0.276
Employed ≥ 20 h/week	250 (20.7)	206 (21.3)	39 (18.3)	5 (19.2)	0.615
Income < 2000 ZAR/month (n = 1203)	727 (60.4)	589 (61.1)	120 (56.3)	18 (69.2)	0.284
Food insecure (mild, moderate, severe)	12 (1.0)	10 (1.0)	2 (0.9)	0 (0.0)	0.1.00
< 5 km from clinic	804 (66.7)	633 (65.5)	153 (71.8)	18 (69.2)	0.195
Current smoker	252 (20.9)	211 (21.8)	37 (17.4)	5 (15.4)	0.272
Current alcohol use	437 (36.3)	365 (37.8)	69 (32.4)	3 (11.5)	**0.009**
Prior diabetes diagnosis (n = 538)	7 (1.3)	6 (1.3)	0 (0)	1 (16.7)	0.082
Uses insulin	2 (28.6)	1 (20)	0 (0)	1 (100)	0.286
Self-reported liver disease	4 (0.3)	3 (0.3)	1 (0.5)	0 (0)	0.587
Self-reported kidney disease	20 (1.7)	14 (1.5)	5 (2.4)	1 (3.9)	0.218
Clinical characteristics and laboratory testing					
Karnofsky score (n = 1368)	87.8 ± 5.9	88.1 ± 5.7	87.3 ± 6.1	84.2 ± 8.6	**0.002**
Body mass index (kg/m^2^)	27.4 ± 6.7	2705 ± 6.5	8.8 ± 6.7	31.3 ± 10.5	
< 25	522 (43.3)	436 (45.0)	79 (37.1)	7 (34.6)	** < 0.001**
25–29.9	322 (26.7)	275 (28.4)	38 (17.8)	9 (34.5)	
≥ 30	363 (30.1)	257 (26.5)	96 (45.1)	10 (38.5)	
Hypertension	255 (21.1)	210 (21.7)	37 (17.4)	15 (30.8)	0.179
Mean arterial pressure (mmHg)	93.3 ± 16.1	92.7 ± 15.3	94.8 ± 17.6	104.4 ± 20.9	** < 0.001**
Hepatitis B (n = 1197)	74 (6.8)	60 (6.3)	13 (5.7)	2 (8.0)	0.898
Anemia (n = 1189)	571 (43.5)	403 (42.2)	100 (48.1)	14 (56.0)	0.131
Hemoglobin (g/dL) (n = 1189)	12.4 ± 2.1	12.4 ± 2.3	12.3 ± 1.9	12.0 ± 2.3	0.316
Women	11.6 ± 1.7	11.6 ± 1.7	11.7 ± 1.6	10.9 ± 1.9	0.194
Men	13.4 ± 2.1	13.6 ± 2.1	13.0 ± 2.1	13.3 ± 1.8	**0.025**
CD4 count (cells/mm^3^) (n = 1201)^a^	353 [209–545]	365 [222–552]	307 [179–496]	324.5 [129–598]	
< 200	281 (23.4)	214 (22.2)	59 (28.1)	8 (30.8)	0.102
200–350	315 (26.2)	247e (25.6)	62 (29.5)	6 (23.1)	
> 350	605 (50.4)	504 (52.2)	89 (42.4)	12 (46.2)	

P-values obtained using chi-square or Fisher's exact test for categorical variables and ANOVA for continuous variables

^a^Median [interquartile range]

Of those with pre-diabetes or diabetes, 73% were overweight or obese compared to 55% of those with normal HbA_1c_ (p < 0.011). Participants with diabetes also had higher MAP (mean 104.4 mmHg) compared to those with pre-diabetes (94.8 mmHg) or normal HbA_1c_ (92.7 mmHg) (p-value < 0.001).

### Diabetes and hyperglycemia screening at HIV testing

Overall, the NNS to identify one newly diagnosed PLHIV at this urban clinic with diabetes was 47 people and the NNS for hyperglycemia was five people (Table 2). The NNS for one new case of hyperglycemia was 14 people among those ≥ 45 years, though only 39% of cases could potentially be identified relying solely on this metric. Using a BMI ≥ 25 kg/m^2^, the NNS to identify one instance of hyperglycemia is 36 people and 73% of cases could be identified.

**Table 2:** Prevalence of elevated hemoglobin A_1c_ and the number needed to screen at the time of HIV testing according to national screening guidelines

Outcome	N_outcome_/N_total_	%	NNS	% hyperglycemic cases identified	% hyperglycemic cases missed
Overall					
Diabetes (HbA_1c_ ≥ 6.5%)	26/1207	2.2	46.5	100	0
Hyperglycemia (HbA_1c_ ≥ 5.7%)	239/1207	19.8	5.1	100	0
Age ≥ 45 years					
Diabetes (HbA_1c_ ≥ 6.5%)	10/143	7.0	14.3	38.5	61.5
Hyperglycemia (HbA_1c_ ≥ 5.7%)	45/143	31.5	3.1	18.8	80.3
BMI ≥ 25 kg/m^2^					
Diabetes (HbA_1c_ ≥ 6.5%)	19/685	2.8	36.0	73.1	26.9
Hyperglycemia (HbA_1c _≥ 5.7%)	153/685	22.3	4.5	64.0	36.0
Age ≥ 45 years and/or BMI ≥ 25 kg/m^2^					
Diabetes (HbA_1c_ ≥ 6.5%)	20/729	2.7	36.5	76.9	23.1
Hyperglycemia (HbA_1c_ ≥ 5.7%)	163/729	22.4	4.5	68.2	31.8

*BMI* body mass index, *HbA1c* hemoglobin A1c, *NNS* number needed to screen

**Fig. 1:**
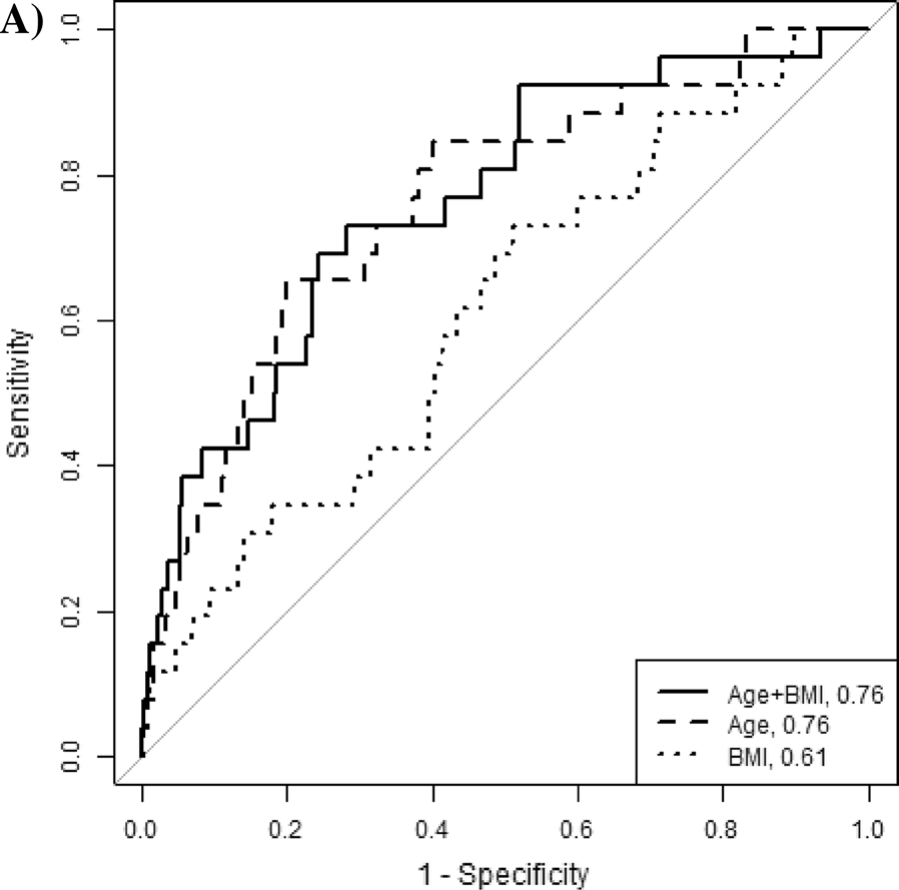

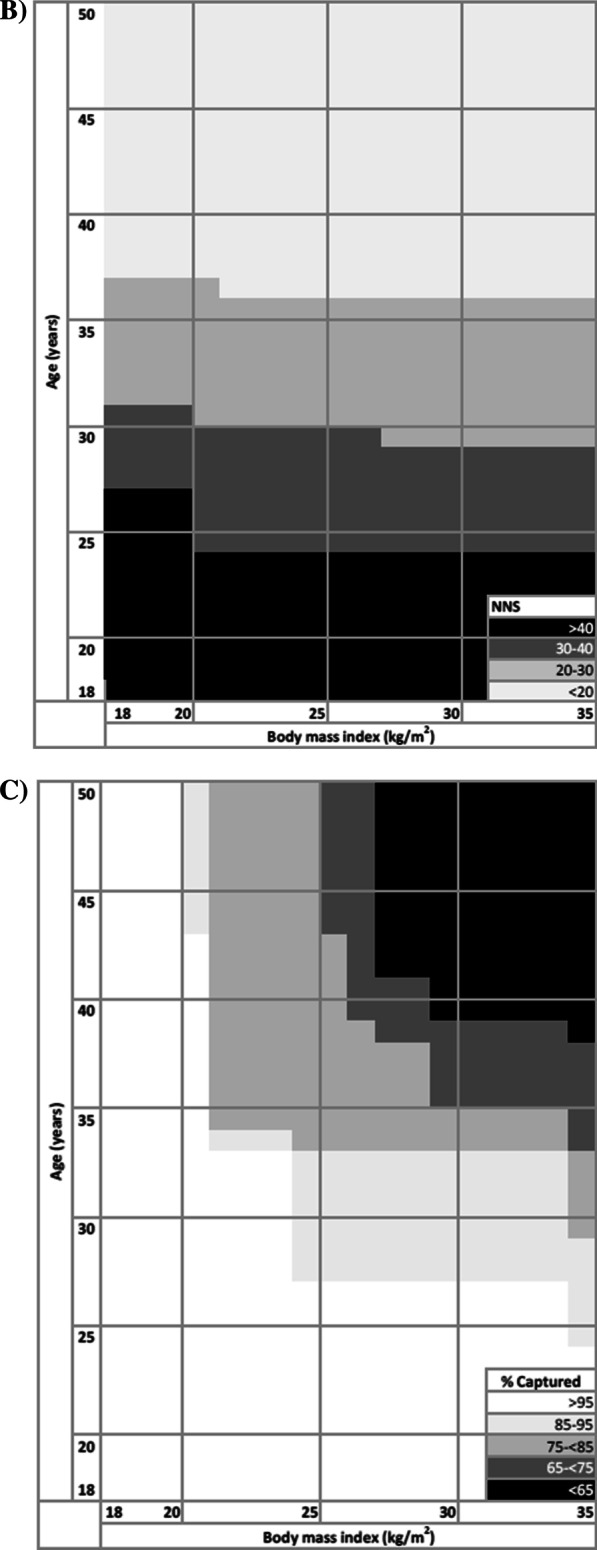
Accuracy (**A**), number needed to screen (**B**), and percent of cases captured (**C**) using age (years) and body mass index as discrete screening criteria for hemoglobin A_1c_ ≥ 6.5%. **A** Distance from the receiver operating characteristic curve to the no discrimination line is maximized for HbA_1c_ ≥ 6.5% at age alone at 38.6 years (specificity: 77.1%, sensitivity: 62.5%, positive likelihood ratio: 2.72), BMI alone at 31.6 kg/m^2^ (specificity: 70.8%, sensitivity: 60.4%, positive likelihood ratio: 2.07). **B** Number need to screen to identify one instance of HbA_1c_ ≥ 6.5%. The lower leftmost black square represents screening the entire population (NNS = 28.5) and the upper rightmost light grey square is the most restrictive screening algorithm represented (NNS = 12.7). **C** Proportion of participants with HbA_1c_ ≥ 6.5% captured. The lower leftmost white square represents screening the entire population (100% captured) and upper rightmost black square is the most restrictive screening algorithm represented (43.8% captured)

Age and BMI were predictive of diabetes with AUCs of 0.76 and 0.61, respectively (Fig. 1A). Using age alone to identify hyperglycemia, the combined sensitivity and specificity were maximized at 39.0 years with a specificity (i.e. the proportion without diabetes who would not be eligible for screening) of 78.5% and sensitivity (i.e. the proportion with diabetes who would be eligible for screening) of 65.4%. The predictive accuracy of BMI alone for hyperglycemia was maximized at 25.5 kg/m^2^ with a specificity of 46.8% and sensitivity of 73.1%.Taking age and BMI together the AUC was 0.76. At 45 years and 25 kg/m^2^, the NNS is 36.5 people (Fig. 1B) and 76.9% of diabetes cases would be captured (Fig. 1C).

### Diabetes and clinical outcomes

The majority of participants initiated ART; 94.8% of those with normal HbA_1c_ initiated ART, 93.9% with moderately elevated HbA_1c_, and 95.2% with high HbA_1c_ (p = 0.811). Among those who initiated ART, there was no difference in the proportion who defaulted, missed ≥ 1 pharmacy refill, or missed ≥ 1 clinic visit by HbA_1c_ level.

In adjusted analyses, there was no difference in the risk of achieving viral suppression (adjusted RR 0.66, 95% CI 0.10–4.36) or receiving a TB diagnosis (adjusted hazard ratio 1.28, 95% CI 0.40–4.1) (Table 3). Median time to death was 182 days (IQR 162–187, n = 37) among participants without diabetes and 139.5 days (IQR 98–181, n = 2) among those with diabetes. The adjusted mortality rate was 1.79-times higher among those with diabetes than those without (95% CI 0.41–7.87).

**Table 3:** Associations of diabetes with HIV outcomes 12 months after HIV diagnosis

	Event/Total_Unexposed,_ N (%)	Event/Total_Exposed,_ N (%)	HR or RR (95% CI)	p-value	aHR or aRR^a^ (95% CI)	p-value
HbA1c ≥ 6.5%						
HIV viral load ≥ 50 copies/mL	136/793 (17.2)	2/13 (15.4)	0.90 (0.25–3.24)	0.868	0.66 (0.10–4.36)	0.666
Hospitalized^b^	73/1181 (6.2)	2/26 (7.7)	1.30 (0.32–5.28)	0.717	1.21 (0.29–5.03)	0.800
Tuberculosis^b^	93/1181 (7.9)	3/26 (11.5)	1.51 (0.48–4.77)	0.483	1.28 (0.40–4.11)	0.680
Died^b^	37/1181 (3.1)	2/26 (7.7)	2.58 (0.62–10.70)	0.192	1.79 (0.41–7.87)	0.443
Retained in care	950/1181 (88.4)	21/26 (80.8)	1.00 (0.83–1.21)	0.928	0.94 (0.78–1.13)	0.512
Default or lost to follow-up	194/1181 (16.4)	3/26 (11.5)	0.70 (0.24–2.05)	0.518	1.01 (0.32–3.20)	0.992
HbA1c ≥ 5.7%						
HIV viral load > 50 copies/mL	106/639 (16.6)	32/167 (19.2)	1.16 (0.81–1.66)	0.428	1.17 (0.81–1.70)	0.413
Hospitalized^b^	63/968 (6.5)	12/239 (5.0)	0.77 (0.42–1.43)	0.414	0.72 (0.39–1.35)	0.227
Tuberculosis^b^	75/968 (7.8)	21/239 (8.8)	1.17 (0.72–1.90)	0.519	1.02 (0.62–1.69)	0.936
Died^b^	29/968 (3.0)	10/239 (4.2)	1.38 (0.67–2.83)	0.382	1.02 (0.47–2.21)	0.962
Retained in care	773/966 (79.9)	198/239 (82.9)	1.03 (0.97–1.11)	0.274	1.03 (0.97–1.10)	0.315
Default or lost to follow-up	166/968 (17.2)	31/239 (13.0)	0.76 (0.53–1.08)	0.125	0.82 (0.59–1.14)	0.243

*aHR* adjusted hazard ratio; *aRR* adjusted risk ratio; *CI* confidence interval; *HR* hazard ratio; *HbA*_*1c﻿*_ hemoglobin A_1c_; *RR* risk ratio

^a^Adjusting for age, sex, body mass index, antiretroviral therapy initiation, anemia, and opportunistic infections excluding tuberculosis when it is the primary exposure of interest and otherwise inclusive of tuberculosis diagnosis

^b^Hazard ratio

Among those with hyperglycemia, there was no difference in the risk of lack of viral suppression, TB, hospitalization, death, retention in care, or loss to follow-up compared to those without hyperglycemia.

## Discussion

In this cohort of newly diagnosed HIV-positive adults in Durban, South Africa, the prevalence of pre-diabetes was high and diabetes was low with few people reporting a prior diabetes diagnosis. Significant risk factors for diabetes included older age, higher BMI, and higher MAP. Diabetes was not associated with 12 month study outcomes. Our data support screening all PLHIV for diabetes or targeting those with a BMI ≥ 25 kg/m^2^ if universal screening is not practical.

The prevalence of diabetes among PLHIV in this cohort was 2.2%, which is similar to observations from other studies of PLHIV in urban South Africa and other sub-Saharan African countries [4, 24–28]. The prevalence of diabetes in the general population in South Africa is estimated to be slightly higher with recent estimates ranging from 5.4% to 10.1% [2, 9, 29]. Risk factors for diabetes in this population included those that are well-established among HIV-uninfected adults and PLHIV in sub-Saharan Africa, such as older age and hypertension [19, 30].

Missed opportunities for diagnosing diabetes remains a major concern in this population. In our cohort, the proportion of people aware of their diabetes was lower than in national prevalence surveys [11, 12]. The Society for Endocrinology, Metabolism, and Diabetes of South Africa recommends diabetes screening for PLHIV at the time of ART initiation or regimen change while South Africa’s national HIV guidelines do not specify recommendations for routine diabetes screening [17]. Inclusion of diabetes screening recommendations in HIV guidelines may help improve uptake.

As in the general population, age and BMI could be used to further increase the yield of diabetes screening by restricting screening to those more likely to have hyperglycemia among PLHIV. We were unable to directly assess if PLHIV were likely to demonstrate elevated HbA_1c_ at relatively lower ages and BMIs compared to HIV-negative adults because of a lack of comparison group. However, data from this cohort suggests screening PLHIV with a BMI ≥ 25 kg/m^2^ could reduce the NNS while capturing most diabetes cases. The AUC suggests the optimal age threshold is lower than guideline of ≥ 45 years for HIV-negative adults, which is supported by research showing PLHIV are more likely to develop diabetes at younger ages [31, 32].

Our study contributes to the literature suggesting the risk of active TB among PLHIV is not substantially elevated in the presence of diabetes, in contrast to the threefold elevated risk of TB among people without HIV infection [34]. Of three studies examining the association of diabetes with active TB among HIV-infected adults, two found an association with higher odds of TB (n = 232, aOR 4.7, 95% CI 1.1–20.8; n = 521, aOR 2.4, 95% CI 1.0–5.9), and one found no effect (n = 382, aOR 0.14, 95% CI 0.01–1.81) [5–7]. In this cohort, ascertainment of TB cases over the 12 month study period was thorough and statistical significance was not achieved.

The presence of multiple morbidities complicates patient care, especially when the diseases differ in pathogenesis and management. Regular clinic visits for ART maintenance are an opportunity for non-communicable disease care. In South Africa, PLHIV on maintenance therapy can have better managed diabetes than the general population, but evidence is mixed [14, 28, 35–37]. In this cohort, there was no significant difference in ART initiation or maintenance, and time to death was elevated but not statistically significantly among with diabetes, possibly due to lack of statistical power. In Brazil and the United States, risk of death among PLHIV was 2–3-times higher for diabetic patients [38, 39]. As testing and diagnosis of diabetes and other non-communicable diseases increase for PLHIV, it will be important to consider how to optimize care for patients and clinics and to study the cost-effectiveness of different approaches to integrated care.

Our study had several strengths and limitations. Our study cohort was large and representative of the township population at risk for HIV. We comprehensively ascertained HIV outcomes by calling the participant and/or family member, and reviewing hospital records, pharmacy records, and national registries. We estimated RRs, which are more consistent, conservative, and interpretable than odds ratios, and used time-to-event analyses where relevant to not introduce bias. However, the prevalence of diabetes was low limiting our ability to detect true associations. Also, we relied on a single point-of-care HbA_1c_ test to screen for diabetes, whereas guidelines recommend two lab-based HbA_1c_ tests several weeks apart [19, 40–44]. Point-of-care HbA_1c_ tests are not standardized, require regular calibration, and therefore are not recommended as diagnostic tests by the WHO or ADA. In prior validation diagnostic studies, A1cNow® + had excellent correlation (> 90%) with gold standard high performance liquid chromatography HbA_1c_, 82% specificity, 100% sensitivity, but moderately overestimated HbA_1c_ levels (mean difference 0.2–0.3% units) [45, 46]. The coefficients of variation (2.7–2.9%) were reasonable for a point-of-care test, although not below the recommended level of < 2% [47]. Additionally, HbA_1c_ may moderately overestimate diabetes among PLHIV especially with concurrent iron deficiency and therefore our estimate of the prevalence of diabetes may be high, although we adjusted for anemia in outcome analyses [19, 40–44]. Lastly, all major risk factors for diabetes were not ascertained in this cohort; therefore, we could not test for associations with all potential risk factors.

## Conclusions

In summary, in urban South Africa hyperglycemia was common among adults testing positive for HIV in an outpatient setting. Diabetes prevalence was lower than the general population, but the majority reported no prior diabetes diagnosis indicating an opportunity for disease detection. The NNS to identify one new case of hyperglycemia was low overall and could be further reduced using age or BMI. Further research is needed to refine diabetes screening guidelines among PLHIV using other methods of diabetes testing, assess the impact of diabetes rapid diagnosis and treatment on HIV outcomes, and optimize integrated chronic disease care.

## Data Availability

The datasets used and analyzed during the current study are available from the corresponding author on reasonable request.

## Abbreviations

ADA: American diabetes association
ART: Antiretroviral therapy
AUC: Area under the curve
BMI: Body mass index
HbA_1c_: Hemoglobin A_1c_
MAP: Mean arterial pressure
NNS: Number needed to screen
OR: Odds ratio
PLHIV: People living with HIV
ROC: Receiver operating characteristic
RR: Risk ratio
TB: Tuberculosis
WHO: World Health Organization

## References

[1] GBD 2016 Causes of Death Collaborators. Global, regional, and national age-sex specific mortality for 264 causes of death, 1980–2016: a systematic analysis for the Global Burden of Disease Study 2016. Lancet. 2017;390(10100):1151–1210. 10.1016/S0140-6736(17)32152-9 10.1016/S0140-6736(17)32152-9 PMC5605883 28919116

[2] IDF Diabetes Atlas. International Diabetes Federation. 8th edn. 2017. Available at: https://www.diabetesatlas.org

[3] Nduka CU, Stranges S, Kimani PK, Sarki AM, Uthman OA (2017). Is there sufficient evidence for a causal association between antiretroviral therapy and diabetes in HIV-infected patients? A meta-analysis. Diabetes Metab Res Rev.

[4] Prioreschi A, Munthali RJ, Soepnel L (2017). Incidence and prevalence of type 2 diabetes mellitus with HIV infection in Africa: a systematic review and meta-analysis. BMJ Open.

[5] Boillat-Blanco N, Ramaiya KL, Mganga M (2016). Transient hyperglycemia in patients with tuberculosis in tanzania: implications for diabetes screening algorithms. J Infect Dis.

[6] Faurholt-Jepsen D, Range N, Praygod G (2011). Diabetes is a risk factor for pulmonary tuberculosis: a case-control study from Mwanza, Tanzania. PLoS ONE.

[7] Oni T, Berkowitz N, Kubjane M, Goliath R, Levitt NS, Wilkinson RJ (2017). Trilateral overlap of tuberculosis, diabetes and HIV-1 in a high-burden African setting: implications for TB control. Eur Respir J..

[8] Hall V, Thomsen RW, Henriksen O, Lohse N (2011). Diabetes in Sub Saharan Africa 1999–2011: epidemiology and public health implications. A systematic review. BMC Public Health.

[9] Stokes A, Berry KM, Mchiza Z (2017). Prevalence and unmet need for diabetes care across the care continuum in a national sample of South African adults: evidence from the SANHANES-1, 2011–2012. PLoS ONE.

[10] Mortality and causes of death in South Africa, 2016: Findings from death notification. Statistics South Africa, 2018.

[11] Hird TR, Pirie FJ, Esterhuizen TM (2016). Burden of diabetes and first evidence for the utility of hba1c for diagnosis and detection of diabetes in urban black South Africans: the Durban diabetes study. PLoS ONE.

[12] Beagley J, Guariguata L, Weil C, Motala AA (2014). Global estimates of undiagnosed diabetes in adults. Diabetes Res Clin Pract.

[13] Njuguna B, Vorkoper S, Patel P (2018). Models of integration of HIV and noncommunicable disease care in sub-Saharan Africa: lessons learned and evidence gaps. AIDS.

[14] Chronic care of HIV and noncommunicable diseases: how to leverage the HIV experience. UNAIDS, 2011. Available at: http://www.unaids.org/sites/default/files/media_asset/20110526_JC2145_Chronic_care_of_HIV_0.pdf

[15] The South African antiretroviral treatment guidelines. 2017.

[16] Knaebel J, Irvin BR, Xie CZ (2013). Accuracy and clinical utility of a point-of-care HbA1c testing device. Postgrad Med.

[17] SEMDSA Type 2 Diabetes Guidelines Expert Committee. The 2017 SEMDSA guideline for the management of type 2 Diabetes. JEMDSA. 2017;22(1):S1–S196.

[18] Use of Glycated Haemoglobin (HbA1c) in the diagnosis of diabetes mellitus: abbreviated report of a WHO consultation. World Health Organization, 2011. 26158184

[19] 2. Classification and diagnosis of diabetes. American Diabetes Association. Diabetes Care. 2018;41(Suppl 1):S13–S27. 10.2337/dc18-S002 29222373

[20] Haemoglobin concentrations for the diagnosis of anaemia and assessment of severity. Vitamin and Mineral Nutrition Information System. World Health Organization, 2011 (WHO/NMH/NHD/MNM/11.1).

[21] Coates J, Swindale A, Biliinsky P. Household Food Insecurity Access Scale (HFIAS) for measurement of food access: indicator guide (v 3). Food and Nutrition Technical Assistance Project, Academy for Educational Development, 2007.

[22] Brown LD, Cai TT, Dasgupta A (2001). Interval estimation for a binomial proportion. Stat Sci.

[23] Youden WJ (1950). Index for rating diagnostic tests. Cancer.

[24] Dave JA, Lambert EV, Badri M, West S, Maartens G, Levitt NS (2011). Effect of nonnucleoside reverse transcriptase inhibitor-based antiretroviral therapy on dysglycemia and insulin sensitivity in South African HIV-infected patients. J Acquir Immune Defic Syndr.

[25] Oni T, Youngblood E, Boulle A, McGrath N, Wilkinson RJ, Levitt NS (2015). Patterns of HIV, TB, and non-communicable disease multi-morbidity in peri-urban South Africa- a cross sectional study. BMC Infect Dis.

[26] Magodoro IM, Esterhuizen TM, Chivese T (2016). A cross-sectional, facility based study of comorbid non-communicable diseases among adults living with HIV infection in Zimbabwe. BMC Res Notes.

[27] Mathabire Rücker SC, Tayea A, Bitilinyu-Bangoh J (2018). High rates of hypertension, diabetes, elevated low-density lipoprotein cholesterol, and cardiovascular disease risk factors in HIV-infected patients in Malawi. AIDS.

[28] Manne-Goehler J, Montana L, Gómez-Olivé FX (2017). The ART advantage: health care utilization for diabetes and hypertension in Rural South Africa. J Acquir Immune Defic Syndr.

[29] Bailey SL, Ayles H, Beyers N (2016). Diabetes mellitus in Zambia and the Western Cape province of South Africa: prevalence, risk factors, diagnosis and management. Diabetes Res Clin Pract.

[30] Njuguna B, Kiplagat J, Bloomfield GS, Pastakia SD, Vedanthan R, Koethe JR (2018). Prevalence, risk factors, and pathophysiology of dysglycemia among people living with HIV in Sub-Saharan Africa. J Diabetes Res.

[31] Butt AA, McGinnis K, Rodriguez-Barradas MC (2009). HIV infection and the risk of diabetes mellitus. AIDS.

[32] Guaraldi G, Orlando G, Zona S (2011). Premature age-related comorbidities among HIV-infected persons compared with the general population. Clin Infect Dis.

[33] Hernandez-Romieu AC, Garg S, Rosenberg ES, Thompson-Paul AM, Skarbinski J (2017). Is diabetes prevalence higher among HIV-infected individuals compared with the general population? Evidence from MMP and NHANES 2009–2010. BMJ Open Diabetes Res Care.

[34] Jeon CY, Murray MB (2008). Diabetes mellitus increases the risk of active tuberculosis: a systematic review of 13 observational studies. PLoS Med.

[35] Chang AY, Gómez-Olivé FX, Manne-Goehler J (2019). Multimorbidity and care for hypertension, diabetes and HIV among older adults in rural South Africa. Bull World Health Organ.

[36] Nugent R, Barnabas RV, Golovaty I (2018). Costs and cost-effectiveness of HIV/noncommunicable disease integration in Africa: from theory to practice. AIDS.

[37] Manne-Goehler J, Siedner MJ, Montana L (2019). Hypertension and diabetes control along the HIV care cascade in rural South Africa. J Int AIDS Soc.

[38] Moreira RC, Pacheco AG, Paula A (2016). Diabetes mellitus is associated with increased death rates among HIV-infected patients in Rio de Janeiro Brazil. AIDS Res Hum Retroviruses.

[39] Park J, Zuñiga JA, García AA (2019). Diabetes negatively impacts the ten-year survival rates of people living with HIV. Int J STD AIDS.

[40] Kim PS, Woods C, Georgoff P (2009). A1C underestimates glycemia in HIV infection. Diabetes Care.

[41] Eckhardt BJ, Holzman RS, Kwan CK, Baghdadi J, Aberg JA (2012). Glycated Hemoglobin A(1c) as screening for diabetes mellitus in HIV-infected individuals. AIDS Patient Care STDS.

[42] Glesby MJ, Hoover DR, Shi Q (2010). Glycated haemoglobin in diabetic women with and without HIV infection: data from the Women's Interagency HIV Study. Antivir Ther.

[43] Slama L, Palella FJ, Abraham AG (2014). Inaccuracy of haemoglobin A1c among HIV-infected men: effects of CD4 cell count, antiretroviral therapies and haematological parameters. J Antimicrob Chemother.

[44] Coban E, Ozdogan M, Timuragaoglu A (2004). Effect of iron deficiency anemia on the levels of hemoglobin A1c in nondiabetic patients. Acta Haematol.

[45] Strauss SM, Rosedale M, Pesce MA (2014). Point-of-care HbA1c testing with the A1cNow test kit in general practice dental clinics: a pilot study involving its accuracy and practical issues in its use. Point Care.

[46] Ginde AA, Cagliero E, Nathan DM, Camargo CA (2008). Point-of-care glucose and hemoglobin A1c in emergency department patients without known diabetes: implications for opportunistic screening. Acad Emerg Med.

[47] Hirst JA, McLellan JH, Price CP (2017). Performance of point-of-care HbA1c test devices: implications for use in clinical practice - a systematic review and meta-analysis. Clin Chem Lab Med.

